# Complete Genome Sequence of the Attenuated *Corynebacterium pseudotuberculosis* Strain T1

**DOI:** 10.1128/genomeA.00947-16

**Published:** 2016-09-08

**Authors:** Sintia Almeida, Dan Loureiro, Ricardo W. Portela, Diego C. B. Mariano, Thiago J. Sousa, Felipe L. Pereira, Fernanda A. Dorella, Alex F. Carvalho, Lilia F. Moura-Costa, Carlos A. G. Leal, Henrique C. Figueiredo, Roberto Meyer, Vasco Azevedo

**Affiliations:** a Laboratory of Cellular and Molecular Genetics, Federal University of Minas Gerais, Belo Horizonte, Minas Gerais, Brazil; b Laboratory of Immunology and Molecular Biology, Federal University of Bahia, Salvador, Bahia, Brazil; c Aquacen, National Reference Laboratory for Aquatic Animal Diseases of Ministry of Fisheries and Aquaculture, Veterinary School, Federal University of Minas Gerais, Belo Horizonte, Minas Gerais, Brazil

## Abstract

We present here the genome sequence of the attenuated *Corynebacterium pseudotuberculosis* strain T1. The sequencing was performed with an Ion Torrent Personal Genome Machine platform. The genome is a circular chromosome of 2,337,201 bp, with a G+C content of 52.85% and a total of 2,125 coding sequences (CDSs), 12 rRNAs, 49 tRNAs, and 24 pseudogenes.

## GENOME ANNOUNCEMENT

*Corynebacterium pseudotuberculosis* is the etiologic agent of caseous lymphadenitis, a chronic disease that affects small ruminants worldwide. *C. pseudotuberculosis* is a Gram-positive bacterium that belongs to the *Corynebacterium*, *Mycobacterium*, *Nocardia*, and *Rhodococcus* (CMNR) group (1). Here, we present the complete genome sequence of *C. pseudotuberculosis* strain T1. This strain was isolated from a goat lymph node in Bahia State, Brazil, and belongs to *C. pseudotuberculosis* bv. ovis; after several passages in culture, it was considered to present low virulence and was used as an attenuated immunogen in goats, producing 33.3% protection against caseous lymphadenitis clinical signs (2). It was described that the secreted/excreted antigens of the T1 strain showed 89% sensitivity and 99% specificity in the detection of specific anti-*C. pseudotuberculosis* IgG antibodies in sheep (1). Later, it was found that these T1 strain antigens were able to stimulate the production of gamma interferon by peripheral blood mononuclear cells of goats and sheep infected with the bacteria (3). The T1 strain could be used as an antigenic model for the detection of specific anti-*C. pseudotuberculosis* IgM antibodies in sheep (4).

The genome was sequenced using the Ion Torrent Personal Genome Machine (PGM) system, a 200-bp-fragment library kit, and a coverage of 110-fold. The quality of the reads was analyzed using the FastQC software (http://www.bioinformatics.babraham.ac.uk/projects/fastqc), and *de novo* assembly was performed using Newbler 2.9 (Roche, USA). The assembly process produced seven contigs with an *N*_50_ value of 367,637 bp. The contigs were oriented and positioned based on an optical map. The optical mapping system measures the lengths of DNA fragments after digestion with restriction enzymes. This high-resolution technique can generate ordered maps of whole genomes and can also be used in the discrimination of closely related bacterial strains (5). Last, the Argus MapSolver software (OpGen, Inc., Gaithersburg, MD) was employed to import the DNA sequence and convert to *in silico* map data. For adjacent contigs with overlapping edges, SIMBA (http://ufmg-simba.sourceforge.net) was used. Repetitive regions were mapped with the CLC Genomics Workbench 7.0 software from Qiagen, USA (CLC bio), using as a reference the genome of *C. pseudotuberculosis* strain 1002. The gap-filling process was done with SIMBA (http://ufmg-simba.sourceforge.net), CLC Genomics Workbench 7.0, and in-house scripts. Automatic annotation was performed by transferring information from a curated database using in-house scripts. Genes encoding tRNAs, rRNAs, and some coding sequences (CDSs) that were absent following the transfer by in-house scripts were predicted using RAST (http://rast.nmpdr.org). All CDSs were manually curated using the Artemis software (6) and the UniProt database (http://www.uniprot.org).

The complete genome of *C. pseudotuberculosis* T1 includes one circular chromosome with a length of 2,337,201 bp, a G+C content of 52.85%, and a total of 2,125 CDSs, 12 rRNAs (5S, 16S, and 23S), 49 tRNAs, and 24 pseudogenes.

### Accession number(s).

The complete genome sequence has been deposited in GenBank under the accession no. CP015100.
